# Perceived consequences and worries among youth in Norway during the COVID-19 pandemic lockdown

**DOI:** 10.1177/1403494821993714

**Published:** 2021-03-01

**Authors:** Stine Lehmann, Jens Christoffer Skogen, Ellen Haug, Silje Mæland, Lars Thore Fadnes, Gro Mjeldheim Sandal, Mari Hysing, Ragnhild Bjørknes

**Affiliations:** 1 Department of Health Promotion and Development, University of Bergen, Norway; 2 Department of Health Promotion, Norwegian Institute of Public Health, Norway; 3 Alcohol and Drug Research Western Norway, Stavanger University Hospital, Norway; 4 Department of Public Health, University of Stavanger, Norway; 5 NLA University College, Bergen, Norway, 6Department of Global Public Health and Primary Care, University of Bergen, Norway; 6 Department of Global Public Health and Primary Care, University of Bergen, Norway; 7 Research Unit for General Practice in Bergen, Norwegian Research Center NORCE, Norway; 8 Bergen Addiction Research, Haukeland University Hospital, Bergen, Norway; 9 Department of Psychosocial Science, University of Bergen, Norway

**Keywords:** Adolescent, pandemics, sleep, family relations, socioeconomic status, ethnicity

## Abstract

**Aims:** To examine perceived consequences for everyday life, learning outcomes, family relations, sleep problems and worries for infection, for friends and their future, among youth aged 12–19 years during weeks 7 to 9 of the COVID-19 lockdown in Norway. We examine variations by age, gender, socioeconomic status and country of birth. **Methods:** Youth within the municipality of Bergen were invited via SMS to participate in a 15-minute online survey. A total of 2997 (40%) youths participated. The mean age was 17 years (standard deviation 1.7). **Results:** Overall, 28% reported feeling somewhat to a lot impacted by schools closing, 63% reported learning less. In total, 62% reported improvement of everyday life. The youth’s situation in their family was worse for 13%. Regarding sleep problems, 19% reported difficulties initiating and maintaining sleep, 12% had more nightmares, while 90% reported later bedtime and rise time. Seven per cent worried about getting infected, while 53% worried about infection among family members. A total of 19% worried that the outbreak would lead to a more difficult future, and 32%worried that friends were facing a difficult situation at home. Perceived consequences and worries related to the lockdown varied across sociodemographic groups. ***Conclusions*: The perceived consequences and degree of worries varied by age, gender, socioeconomic status and to a certain degree country of birth. Girls, older youth, youth with lower socioeconomic status and with a migrant background from developing countries seemed to experience the lockdown as more difficult, and thereby possibly accentuating the need for services in these groups.**

## Background

The World Health Organization declared the COVID-19 outbreak a pandemic on 11 March 2020 [1]. On 12 March Norwegian authorities announced a national lockdown to suppress the outbreak [2]. The strategies implemented included social distancing measures, with closing of schools and organised leisure activities. Health and social services for youth and families were closed or substantially reduced. This resulted in limited access to services for children in need of follow-up during this period [3, 4].

This lockdown yielded dramatic changes to the lives of youth and their families in the following weeks. Ad-hoc home schooling was implemented, and many parents became directly involved, by assisting with home schooling routines. Social support and time spent with friends and teachers decreased, and around half of the parents were working from home [5]. Inevitably, families were spending more time together. Youth were also exposed to media coverage of a potentially life-threatening disease, and warnings of enormous economic consequences for society. However, information that COVID-19 symptoms appeared less severe among youth were available early [6]. Still, they could be infectious and were thereby seen as a threat for older family members.

The level of worries and stress were raised in the world due to the COVID-19 pandemic [7], especially among women, youth and students. These findings are in line with results from Norway [8]. UNICEF has raised concern that schools closure measures may cause mental health problems and psychological distress among children [9]. A recent rapid review [10] identified three studies with parents reporting adverse psychological effects of physical distancing during the COVID-19 outbreak on their children, especially an increase in depressive symptoms, anxiety, irritability, boredom and stress. Among adults, delayed sleep phase and poorer sleep quality have been reported during the COVID-19 outbreak [11], with similar patterns among preschool children [12]. Research on sleep for adolescents during the pandemic has been called for [13].

Meanwhile, the Norwegian context may also have protected youth from some of the stressors related to the outbreak. The lockdown was not complete but was an effort to balance between regulation and advice, prioritising health. During the first weeks, people’s trust in government, parliament and the health authorities increased [14]. Christensen and Lægreid [15] concluded that Norway performed well in handling the crises, due to competent politicians and reliable bureaucracy, a strong public sector and a well-developed welfare state. On the individual level, youth entered the crisis with overall good health, strong family relations, high levels of wellbeing and school satisfaction, and relatively high family wealth compared to other European countries [16–18]. These factors are considered important resources for resilient outcomes for youth [19, 20].

However, not much is known about the impact of pandemics accompanied by severe restrictions such as schools closing for youth. The present paper focuses on perceived consequences and worries due to the lockdown caused by COVID-19, among youth in Norway.

### Aims

During weeks 7 to 9 of the COVID-19 lockdown: To examine perceived consequences of lockdown for everyday life, learning outcomes, family relations, sleep; and worries about contamination, for friends and their future, among youth aged 12–19 years. We examined variations by age, gender, socioeconomic status and country of birth.

## Methods

### Design, procedure, and participants

COVID-19 Young is an epidemiological study of youth aged 12–19 years within the municipality of Bergen. It comprises two samples: cohort 1 were youth aged 12–15 years in Bergen municipality whose parents had participated in the study Bergen in Change and consented that their child could be invited to the study. These parents were more often women (Cramérs V: 0.069, *P*<0.001), older (Cramérs V: 0.092, *P*<0.001), had higher educational attainment (Cramérs V: 0.155, *P*<0.001) and household income (Cohen’s D: 0.19, *P*<0.001) and had less often shared residence for the child (Cramérs V: –0.054, *P*=0.006) when compared to non-consenting parents. The differences observed ranged between very small and small effect sizes. On consent, parents provided contact information for the youth. A total of 1565 youth were contacted. Cohort 2 was youth aged 16–19 years, attending high schools in Bergen municipality. The county provided phone numbers from their contact registers. A total of 5947 youth was contacted in cohort 2.

The data collection started on 27 April, during the 7th week of lockdown, and closed on 11 May. The invitation procedures were the same for cohorts 1 and 2. Youth were recruited via SMS and a link to a secure online platform, containing an information letter and a 15-minute online survey. Two SMS reminders were given. Participants were included in a lottery for a new cell phone. A total of 7512 youth was invited to participate. Of these, 843 (54%) in cohort 1 and 2154 (36%) in cohort 2 responded, yielding a total of 2997 (40%) youths completing the survey. The mean age was 17 years (standard deviation (SD) 1.7), 57.7% were girls, and most participants reported living with both parents (77.5%), being born in Norway (93%) and living with siblings (71%).

### Measures

Demographic information was measured by youth self-report on gender, age, country of birth and living arrangements.

Socioeconomic status (SES) was measured by self-rated family affluence for cohort 2 with the following question: ‘How well off do you think your family is compared to others’? For cohort 1, aged 12–15 years, we used parental report on household income as indicator for SES. Parental reported data were not available for cohort 2, while data on self-rated family affluence were not available for cohort 1.

Perceived consequences of the COVID-19 pandemic shutdown were measured by self-report of: being impacted by schools closing; learning outcome; possible positive consequences of schools closing; whether the youth themselves or their family had been infected; and whether they perceived their own family situation as changed, for the better or worse during this period.

Worries due to the COVID-19 pandemic shutdown was measured with youth self-report on: whether the youth worried about COVID-19 infection, for themselves, their family or close ones; whether friends faced a difficult situation in their family during the shutdown; and worried that they themselves would face a harder future as a consequence of the COVID-19 pandemic.

Sleep problems and patterns were assessed with four items: (a) experiencing difficulties initiating and maintaining sleep (DIMS); (b) nightmares more often; (c) later bedtime; and (d) later rise time.

The phrasing of all items may be found in Supplemental Table I.

### Statistical analyses

In the present analyses we excluded all that did not have valid responses on age and gender (*n*=64; 2.1%). For all tables, the following statistics are presented: *n* (%) and *P* values for chi-square test of independence, as well as variable-specific number of missing. We show results stratified by gender (Table I), age groups (Table II), socioeconomic indicators (Table III) and country of birth (Table IV). Due to some missing information on the stratification variables (ranging from *n*=7 to *n*=40), the overall number of valid responses in the tables varies between 2933 and 2893. To retain the maximum level of information, pair-wise deletion was used throughout the analyses.

**Table I.** Description of sample and included variables across gender, *N*=2933.

Variable		Male, *n*=1242^a^	Female, *n*=1691^a^	*P* value^b^	Missing
**Demographic variables**		%	%		*n*
Age, years				0.144	0
	12	6.0	5.1		
	13	7.8	6.1		
	14	6.2	6.4		
	15	8.1	6.6		
	16	22	25		
	17	37	37		
	18 or older	13	14		
Country of birth				0.471	7
	Norway	93	92		
	Other W. country	4.2	4.3		
	Developing country	2.8	3.6		
SES: household income^c^				0.458	9
	<25th percentile	22	25		
	25–75th percentile	49	50		
	>75th percentile	29	25		
SES: self-reported family affluence^d^				<0.001	31
	Worse	8.2	11		
	About the same	62	72		
	Better	30	17		
**Consequences of COVID-19 lockdown**					
Infected, self or others				0.316	0
	Yes	27	25		
Impacted by schools closing				<0.001	51
	Not at all	8.8	5.7		
	A little	41	37		
	Somewhat	26	30		
	A lot	17	17		
	Very much	7.0	10		
Learned				0.760	
	Less	64	63		61
	About the same	25	26		
	More	11	11		
Parts of everyday life improved				0.376	68
	Yes	61	62		
Living with family				<0.001	231
	Better	19	21		
	The same	71	64		
	Worse	10	15		
DIMS				<0.001	238
	Not true	57	43		
	Somewhat true	28	35		
	Very true	14	22		
Nightmares more often					260
	Yes	6.6	16		
Later bedtime				0.180	280
	Yes (<1 h)	48	49		
	Yes (>1 h)	38	39		
Later rise time				<0.001	250
	Yes (<1 h)	57	60		
	Yes (>1 h)	28	32		
**Worries due to COVID-19 lockdown**					
Worried about getting infected self				<0.001	93
	Not true	61	49		
	Somewhat true	34	42		
	Very true	4.4	8.9		
Worried about family getting infected				<0.001	91
	Not true	16	7.9		
	Somewhat true	41	33		
	Very true	44	59		
Worry for a more difficult future				<0.001	94
	Not true	52	42		
	Somewhat true	32	36		
	Very true	16	22		
Worried for friend’s family situation				<0.001	227
	Yes	26	36		

DIMS: difficulties initiating and maintaining sleep; SES: socioeconomic status.

a Statistics presented: %.

b Statistical tests performed: chi-square test of independence.

c Only applicable for cohort 1.

d Only applicable for cohort 2.

<25th percentile: <1 m NOK; 25–75th percentile: 1–1.5 m NOK; >75th percentile: >1.5 m NOK.

**Table II.** Perceived consequences and worries across age groups, *N*=2933.

Variable		12–15 years, *N*=759^a^	16–19 years, *N*=2174^a^	*P* value^b^	Missing
**Consequences of COVID-19 lockdown**		%	%		n
Infected, self or others				0.164	0
	Yes	25	27		
Affected by schools closing				<0.001	51
	Not at all	6.9	7.0		
	A little	48	36		
	Somewhat	27	29		
	A lot	13	18		
	Very much	4.1	11		
Learned				<0.001	61
	Less	54	67		
	About the same	33	23		
	More	13	10		
Everyday life improved				0.220	68
	Yes	64	61		
Living with family				0.001	231
	Better	23	19		
	The same	67	67		
	Worse	9.6	14		
DIMS				<0.001	238
	Not true	59	45		
	Somewhat true	28	33		
	Very true	13	21		
Nightmares more often				0.002	260
	Yes	9.5	13		
Later bedtime				<0.001	290
	Yes (<1 h)	59	44		
	Yes (>1 h)	24	44		
Later rise time				<0.001	250
	Yes (<1 h)	64	57		
	Yes (>1 h)	21	34		
**Worries due to COVID-19 lockdown**					
Worried about getting infected self				<0.001	93
	Not true	59	52		
	Somewhat true	36	40		
	Very true	4.8	7.8		
Worried about family getting infected				0.001	91
	Not true	14	10		
	Somewhat true	39	35		
	Very true	48	55		
Worry for a more difficult future				<0.001	94
	Not true	56	42		
	Somewhat true	34	35		
	Very true	9.7	23		
Worried for friend’s family situation				0.415	227
	Yes	30	32		

DIMS: difficulties initiating and maintaining sleep.

a Statistics presented: %.

b Statistical tests performed: chi-square test of independence.

**Table III.** Perceived consequences and worries across socioeconomic indicators, *N*=2893.

Variable		Cohort 1 (*N*=802)				Cohort 2 (*N*=2091)				Missing	
		<25th percentile, *N*=189^a^	25–75th percentile, *N*=396^a^	>75th percentile, N=217^a^	*P* value^b^	Worse, *N*=210^a^	About the same, *N*=1422^a^	Better, *N*=459^a^	*P* value^b^	C1	C2
**Consequences of COVID-19 lockdown**											
		%	%	%		%	%	%		*n*	*n*
Infected, self or others					0.051				0.474	0	0
	Yes	24	22	31		27	25	28			
Affected by schools closing					0.396				<0.001	10	13
	Not at all	5.3	8.2	5.6		4.8	6.8	9.0			
	A little	51	48	43		25	36	40			
	Somewhat	24	26	33		29	30	24			
	A lot	16	14	13		20	18	18			
	Very much	4.3	3.6	5.2		21	9.6	9.6			
Learned					0.409				0.026	13	20
	Less	54	52	59		75	66	63			
	About the same	30	35	28		15	24	26			
	More	16	13	13		9.7	9.8	11			
Everyday life improved					0.461				0.153	18	22
	Yes	60	65	63		55	62	62			
Living with family					0.043				<0.001	43	158
	Better	19	26	19		19	17	25			
	The same	66	65	71		51	69	66			
	Worse	14	8.2	9.9		30	13	8.6			
DIMS					0.041				<0.001	45	164
	Not true	51	62	58		25	46	52			
	Somewhat true	30	28	29		34	34	30			
	Very true	19	10	13		41	19	18			
Nightmares more often					0.194				0.002	48	183
	Yes	8.5	8.5	12		20	14	10			
Later bedtime					0.023				0.140	60	200
	Yes (<1 h)	51	60	58		37	46	44			
	Yes (>1 h)	35	22	23		51	42	45			
Later rise time					0.465				0.324	54	167
	Yes (<1 h)	59	63	68		51	57	59			
	Yes (>1 h)	25	22	18		39	34	31			
**Worries due to the COVID-19 lockdown**											
Worried about getting infected					0.192				<0.001	17	47
	Not true	61	57	64		42	51	60			
	Somewhat true	34	37	34		48	41	35			
	Very true	4.9	5.6	1.9		10	8.6	5.1			
Worried about family getting infected					0.803				0.003	17	45
	Not true	14 14		13		11	9.1	12			
	Somewhat true	41	37	42		27	35	40			
	Very true	46	49	45		62	56	48			
Worry for a more difficult future					0.919				<0.001	17	48
	Not true	56	55	58		26	44	43			
	Somewhat true	35	35	32		32	35	37			
	Very true	9.2	11	10		41	21	20			
Worried for friend’s family situation					0.130				<0.001	44	154
	Yes	35	27	33		45	31	31			

DIMS: difficulties initiating and maintaining sleep.

a Statistics presented: %.

b Statistical tests performed: chi-square test of independence.

Cohort 1; parental-reported household income.

Cohort 2; self-reported relative socioeconomic status.

<25th percentile: <1 m NOK; 25–75th percentile: 1–1.5 m NOK; >75th percentile: >1.5 m NOK.

**Table IV.** Perceived consequences and worries across country of birth, *N*=2926.

Variable		Norway, *N*=2707^a^	Other western country, *N*=125^a^	Developing country, *N*=94^a^	*P* value^b^	Missing, *n*
**Consequences of COVID-19 lockdown**		%	%	%		
Infected self, or others					0.228	0
	Yes	26	22	32		
Affected by schools closing					0.046	44
	Not at all	6.9	8.2	8.7		
	A little	39	39	33		
	Somewhat	29	24	21		
	A lot	17	17	20		
	Very much	8.5	11	18		
Learned					0.883	54
	Less	63	61	64		
	About the same	26	26	27		
	More	11	13	8.7		
Everyday life improved					0.124	61
	Yes	62	61	52		
Living with family					0.101	224
	Better	20	27	21		
	The same	67	65	60		
	Worse	13	8.0	19		
						
DIMS					0.158	231
	Not true	49	48	48		
	Somewhat true	32	30	24		
	Very true	19	21	29		
Nightmares more often					0.643	253
	Yes	12	11	13		
Later bedtime					0.715	283
	Yes (<1 h)	49	45	43		
	Yes (>1 h)	38	41	40		
Later rise time					0.004	243
	Yes (<1 h)	60	46	48		
	Yes (>1 h)	30	37	36		
**Worries due to the COVID-19 lockdown**						
Worried about getting infected self					<0.001	86
	Not true	55	50	33		
	Somewhat true	39	41	46		
	Very true	6.5	9.1	21		
Worried about family getting infected					0.136	84
	Not true	11	13	8.9		
	Somewhat true	36	38	26		
	Very true	53	49	66		
Worry for a more difficult future					0.006	87
	Not true	46	45	37		
	Somewhat true	35	29	31		
	Very true	19	26	32		
Worried for friend’s family situation					0.227	220
	Yes	32	25	36		

DIMS: difficulties initiating and maintaining sleep.

a Statistics presented: %.

b Statistical tests performed: chi-square test of independence.

### Ethics

Youth consented to participate by ticking the consent form at the start of the survey. The Regional Committee for Medical and Health Research Ethics, Western Norway approved the study (project number 131560).

## Results

### COVID-19-related consequences and worries

For the total sample, 54% reported being ‘somewhat’, ‘a lot’ or ‘very much’ impacted by schools getting closed, and 63% reported learning less. In total, 62% reported that parts of everyday life had improved in this period. Calmer days at home (75% of those reporting improvement), more time with their family (63%), more time outdoors (49%) and social contact with more people online (43%) were the most frequently reported improvements. The youth’s own situation in their family was reported as better for 20% of the sample, and worse for 13%. Regarding sleep problems, 19% reported DIMS, 12% had more nightmares, while nine out of 10 reported one hour or more later bedtime and rise time after schools closed. Few of the youths reported that they themselves had been infected (*N*=22; *n*=12 boys and *n*=10 girls).

Seven per cent were worried that they would get infected, while 53% were worried that someone else in their family would get infected. A total of 19% were worried that the outbreak would lead to a more difficult future for themselves, and 32% were worried that some of their friends were facing a difficult situation at home after schools closed.

### Consequences and worries across genders

Table I presents background variables (age, country of birth, household income (cohort 1), relative socioeconomic status (cohort 2) and variations in perceived consequences and worries due to the lockdown across genders. Girls reported being more impacted by schools being closed than boys and a higher proportion reported that living with their family was worse after schools closed. Girls also reported more DIMS, more nightmares and later rise time than boys. Girls reported a higher level of worry compared to boys.

### Consequences and worries across age groups

Table II presents variations across age groups (12–15 years vs. 16–19 years). Older youth reported being more impacted by schools getting closed and learning less. They also more often reported that living with their family was worse after schools closed. Older adolescents reported more DIMS, more nightmares and later bedtime and rise time. Regarding worries, higher age was associated with more worry about themselves or family members getting infected and more worry about a more difficult future for themselves. No other significant differences between the age groups were identified.

### Consequences and worries across SES indicators

Table III presents variations across SES indicators. For cohort 1, youth in families with lower household income reported more often that living with their family was worse, and a higher proportion reported an increase in DIMS, as well as later bedtime after schools closed. For cohort 2, lower relative SES was associated with being more impacted by schools closing, learning less and that living with their family was worse after schools closed. Regarding sleep, DIMS and more nightmares were associated with lower subjective SES. The same was true for worries about themselves or family members getting infected and worry that the outbreak will lead to a more difficult future for them. Youth in the lowest SES group also reported more worry about their friends’ situations at home after the schools closed.

### Consequences and worries across participants’ country of birth

Table IV presents variations across where the participant was born. A higher proportion of participants born outside of Norway reported being impacted by the schools getting closed, with a higher proportion endorsing ‘a lot’ and ‘very much’ among migrant youth born in developing countries. Those born outside of Norway were less likely to report changes in rise time from bed compared those born in Norway. Also, youth born outside of Norway worried more about getting infected themselves and about their own future.

A description of sample and included variables across genders, age groups, socioeconomic indicators and country of birth, including 95% confidence intervals can be found in Supplemental Figures A1–A5.

## Discussion

The current study provides an overview of the perceived consequences and worries of youth from a large municipality in Norway during weeks 7 to 9 of the COVID-19 lockdown. Almost half (46.2%) reported that they were not or only a little impacted by schools being closed. However, over 60% reported learning less than normal in this period. Over 60% of the youth reported that parts of their everyday life had improved after the schools closed. Still, 13% reported that their family situation had become worse. Half of the youth reported more DIMS during this period. Three to four out of 10 reported bedtime and/or rise time one hour later than usual or more, signifying a substantial proportion of youth with delayed sleep phase during the lockdown.

While a little fewer than half of the participants worried about contamination for themselves, almost nine out of 10 were worried about their family being infected. A third of the youth reported that they worried that some of their friends faced a difficult situation at home after the schools closed. A little over half of the youth worried that the COVID-19 outbreak will lead to a more difficult future for themselves. Overall, the findings suggest that a considerable proportion of this sample of youth seemed partly to adjust favourably to the lockdown of Norway. However, one important finding is that the perceived consequences and worries related to the lockdown vary across sociodemographic groups.

Overall, a higher proportion of older youth was impacted by schools closing, reported that they learned less, and experienced a worsened family situation. Older youth also worried more about themselves or close ones being infected by the virus. The reasons for these age-related differences are probably multifactored and interrelated. Youth in the age group from 16 to 19 years are in a transitional phase to adulthood, in which the relative importance of school achievements is higher, and other important life domains such as sports, relationships and potential work changes might be more impacted by the lockdown. In part due to growing cognitive abilities allowing anticipatory thoughts about negative consequences for the future, worries seem to increase with age during this developmental period [21]. School is the source of the most intense and most frequent worries in everyday life for adolescents [21, 22]. This was also evident under the home schooling arrangement during the COVID-19 outbreak. Older youth are also more likely to follow news updates, especially on social media [23], and were more likely to be exposed to news regarding the pandemic, while also being more likely to understand the potential impact of both the pandemic and the lockdown on society. The fact that 67% experienced a lower learning outcome suggests that the home-school arrangements offered were not adequate for the older youths. From a societal and individual perspective, inadequate home-school arrangements may be of concern, as academic achievement is associated with factors such as students’ subjective wellbeing [24] and dropping out of school [25].

Another finding was that a higher proportion of girls reported being impacted by the lockdown, reported a worsened family situation and had worries about themselves or close ones being infected by the COVID-19 virus; and also worried about their friends and their own future. The finding that the lockdown seems to impact girls more negatively might not be surprising, as girls in general are subjected to more symptoms of anxiety and depression than boys [26]. It is also well documented that girls experience more school pressure and have higher levels of subjective health complaints compared to boys [17]. Still, the findings suggest the need for a gender perspective on the consequences of COVID-19 when assessing the long-term impact of the measures and strategies implemented during the pandemic.

Youths from families with lower SES were disproportionally negatively impacted by the outbreak across life domains including learning outcome, their situation in the family and worry about infection. Somewhat surprisingly, we did not find that SES was associated with variations in everyday life improvement during the lockdown. Our finding is in line with a recent study, concluding that under the COVID-19 pandemic, the social gradient in life satisfaction decreased among youth [27]. Still, we did find a social gradient regarding the youths’ situation in their own family, indicating that youth with lower SES experienced a worsening of their family situation. Our results confirm the stated worry that children from low SES families will be especially vulnerable to closed schools [28].

Sleep problems were prevalent among youth during the COVID-19 lockdown, with the expected female preponderance of sleep problems, the social gradient of sleep and increase by age, in accordance with pre-pandemic population-based studies [29]. A delay in sleep phase during this period of the COVID-19 outbreak is in line with previous COVID-19 outbreak studies [30]. A substantial minority reported an increase in nightmares, a sleep problem that often presents because of worry and negative life experiences. Given the importance of sleep for learning and mental health in this developmental phase, follow-up studies should assess the duration of these sleep problems.

Some differences related to country of birth were also observed. Among migrant youth from developing countries, a higher proportion reported being negatively impacted by schools closing, and they worried more about being infected and about their own future. This finding may be related to several aspects, such as the media coverage of more people from ethnic minority groups getting infected than other groups in Norway. Language problems causing less access to information may also have resulted in more uncertainty and worries among ethnic minority families. While guidelines following the crisis in different languages were posted on webpages during the lockdown, the Norwegian government acknowledged the need for more targeted communication to minority populations. Moreover, differences in worries may be associated with socioeconomic and cultural factors [31]. In particular, more immigrants live in crowded houses and with more intergenerational contact. During the pandemic, there has been a strong increase in unemployed immigrants in Norway because of lay-offs [32]. Thus, many immigrant youths live in families experiencing negative consequences of COVID19.

### Limitations

One limitation is that most indicators were based on self-report, and the reliability and validity of many of the items have not been tested. We did not have data on parents’ income for cohort 2, and therefore used self-reported family affluence. However, there are indications that subjective assessments of SES are meaningful measures irrespective of objective family socioeconomic background [33]. Furthermore, the characteristics of the parents for the youngest cohort suggest that this was a selected group of youth, whose parents had higher educational level and household income compared to non-consenting parents. This, together with relatively low response rates, warrants caution in interpreting the findings, as they might not be generalisable, and age group differences must be interpreted with caution. On the other hand, a strength of our study is that the sample is population-based and a probability sample, as opposed to convenience samples. Sleep-related aspects were only assessed in one direction; measuring sleep problems, and not improved sleep quality. One cannot out rule the possibility of improved sleep for some during this period, and this should be assessed in an eventual follow-up study.

## Conclusions

Overall, almost half of the youth reported that they were not or only to a little degree impacted by the schools being closed. Even so, our results show that a higher proportion of girls, older adolescents, youth with low SES and youth born outside of Norway experience negative consequences and worries during the first period of the COVID-19 outbreak when schools were closed to limit the spread of the virus. These results may indicate that the need for services for these groups may be accentuated due to ongoing public health measures during the pandemic, especially if these measures impact the daily life of youth; that is, schools closure and physical distancing. Given that health and child welfare services for youth and families to a certain extent failed in following up youth during the lockdown, attention to access to relevant services for youth in need is called for in coming waves of a pandemic outbreak.

## Supplemental Material

sj-pdf-1-sjp-10.1177_1403494821993714 – Supplemental material for Perceived consequences and worries among youth in Norway during the COVID-19 pandemic lockdown Click here for additional data file. Supplemental material, sj-pdf-1-sjp-10.1177_1403494821993714 for Perceived consequences and worries among youth in Norway during the COVID-19 pandemic lockdown by Stine Lehmann, Jens Christoffer Skogen, Ellen Haug, Silje Mæland, Lars Thore Fadnes, Gro Mjeldheim Sandal, Mari Hysing and Ragnhild Bjørknes in Scandinavian Journal of Public Health
