# Supplementary material for: Perceived consequences and worries among youth in Norway during the COVID-19 pandemic lockdown
Source: Scand J Public Health. 2021 Mar 1;49(7):755–65. doi: 10.1177/1403494821993714 (PMC8521367; doi:10.1177/1403494821993714)
Supplement: sj-pdf-1-sjp-10.1177_1403494821993714 – Supplemental material for Perceived consequences and worries among youth in Norway during the COVID-19 pandemic lockdown [file sj-pdf-1-sjp-10.1177_1403494821993714.pdf]

| | |
|---|---|
| Age, years | How old are you? |
| Country of birth | Where are you born |
| SES: Household income | Parents income (parent-reported) |
| SES: Self-reported family affluence | How well off is your family compared to most other families? |
| **Consequences of COVID-19 lockdown** | |
| Infected, self or others | Have you, or anyone you know been infected by the Corona virus?<br>(6)□No<br>(2)□Yes, me<br>(3)□Yes, someone in my household<br>(4)□Yes, others in my family<br>(5)□Yes, someone outside my family |
| Impacted by schools closing | How much are you impacted by the fact that your school is closed? |
| Learned | During the weeks of homeschooling, do you feel you have learned... |
| Parts of everyday life improved | Do you think parts of your everyday life have improved after the school closed?<br><br>If yes: what has improved?<br>More than one answer is possible.<br>(1)□More time with the family<br>(2)□More time with friends<br>(3)□Parents are more present<br>(4)□Calmer days at home<br>(5)□I / we are doing new things / activities<br>(6)□More time outdoors<br>(7)□Have social contact with more people, online or on mobile (e.g. family, friends)<br>(8)□Grown-ups have been better on digital tools<br>(9)□Other, please note what:_____ |
| Living with family | How are you getting along with your family during this period after school closing?<br>(1)□A lot better<br>(2)□A little better<br>(3)□As before the school closed<br>(4)□A little worse<br>(5)□A lot worse |
| DIMS | We would like to know how you have been sleeping the last couple of weeks, after your school closed:<br>Have you had problems sleeping, or do you wake up frequently during the night? |
| Nightmares more often | After the school closed, have you had nightmares or unpleasant dreams? |
| Later bedtime | After the school closed, have you gone to bed later than usual on weekdays? |
| Later risetime | After the school closed, have you raised from bed later than usual on weekdays? |
| **Worries due to COVID-19 lockdown** | |
| Worried about getting infected self | I’m worried about getting infected myself |
| Worried about family getting infected | I’m worried about someone in my family getting infected |
| Worry for a more difficult future | I’m worried that the outbreak will lead to a more difficult future for me |
| Worried for friend’s family-situation | Are you worried that some of your friends are struggling at home with their family now while the school is closed? |

Supplementary Table 1. Items and response alternatives  for each variable included in the analyses

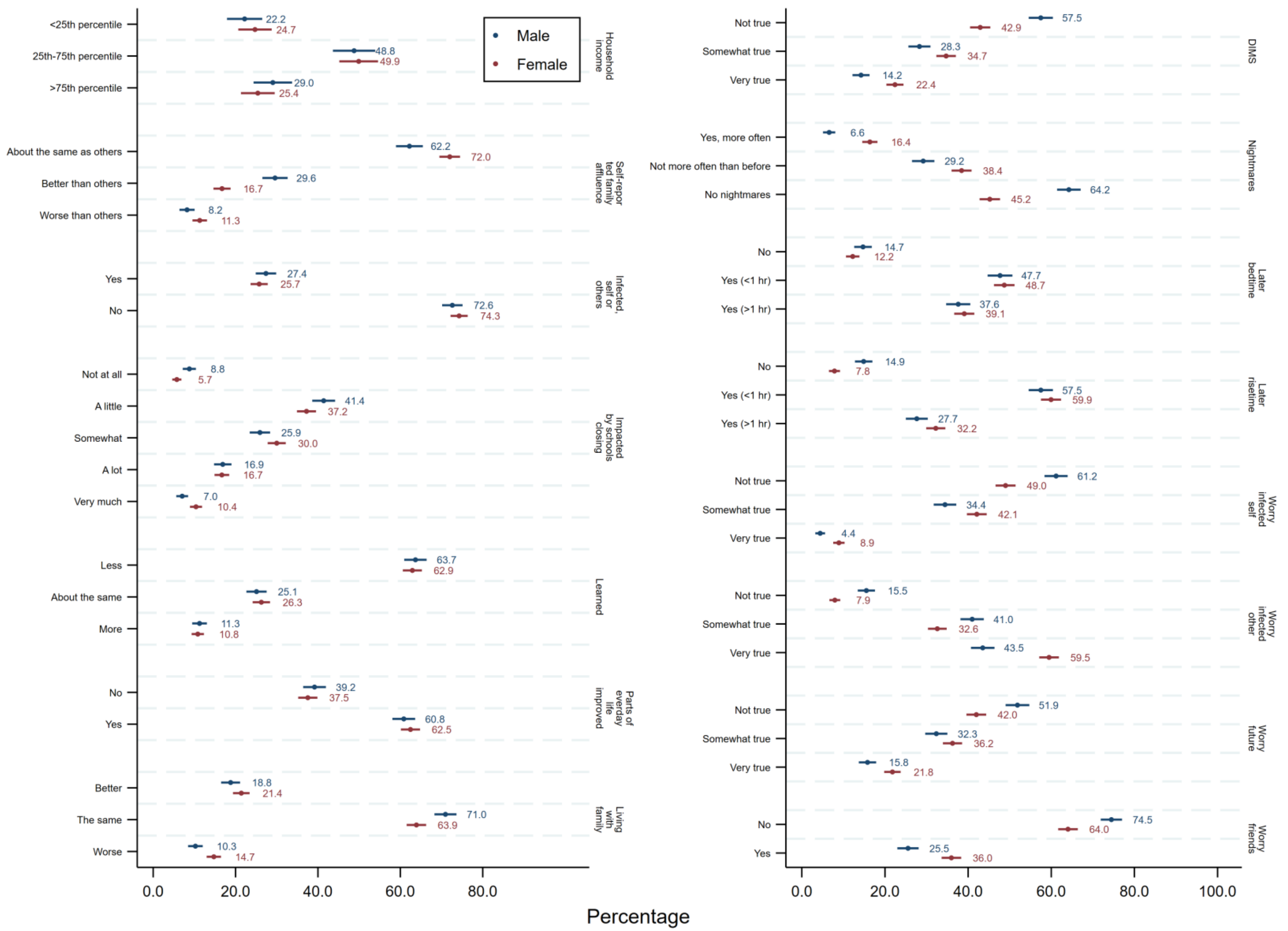


Supplementary Figure A1: Description of sample and included variables across gender. N=2933. Bars denote 95% confidence interval.

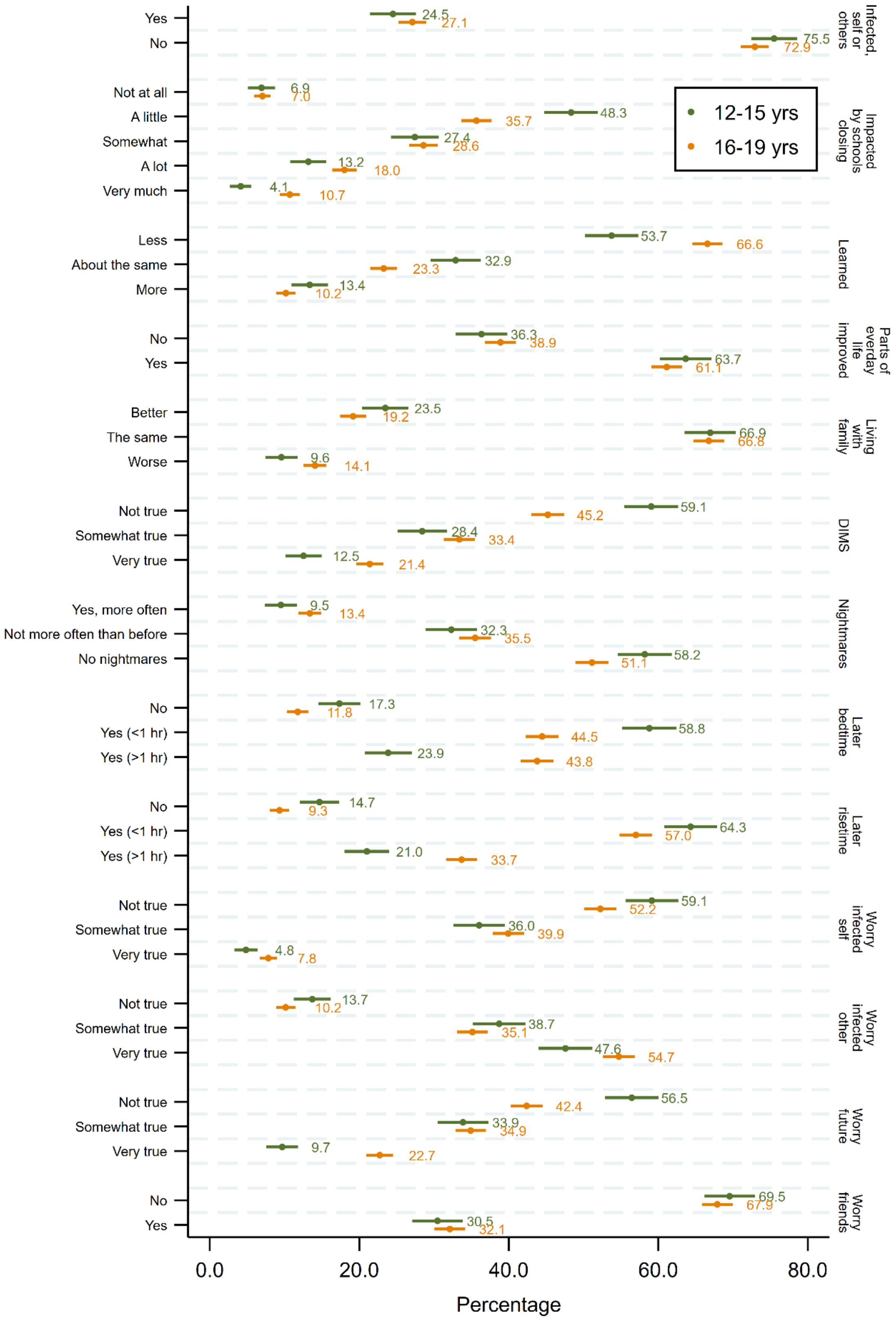


Supplementary Figure A2: Perceived consequences and worries across age groups. N=2933. Bars denote 95% confidence intervals.

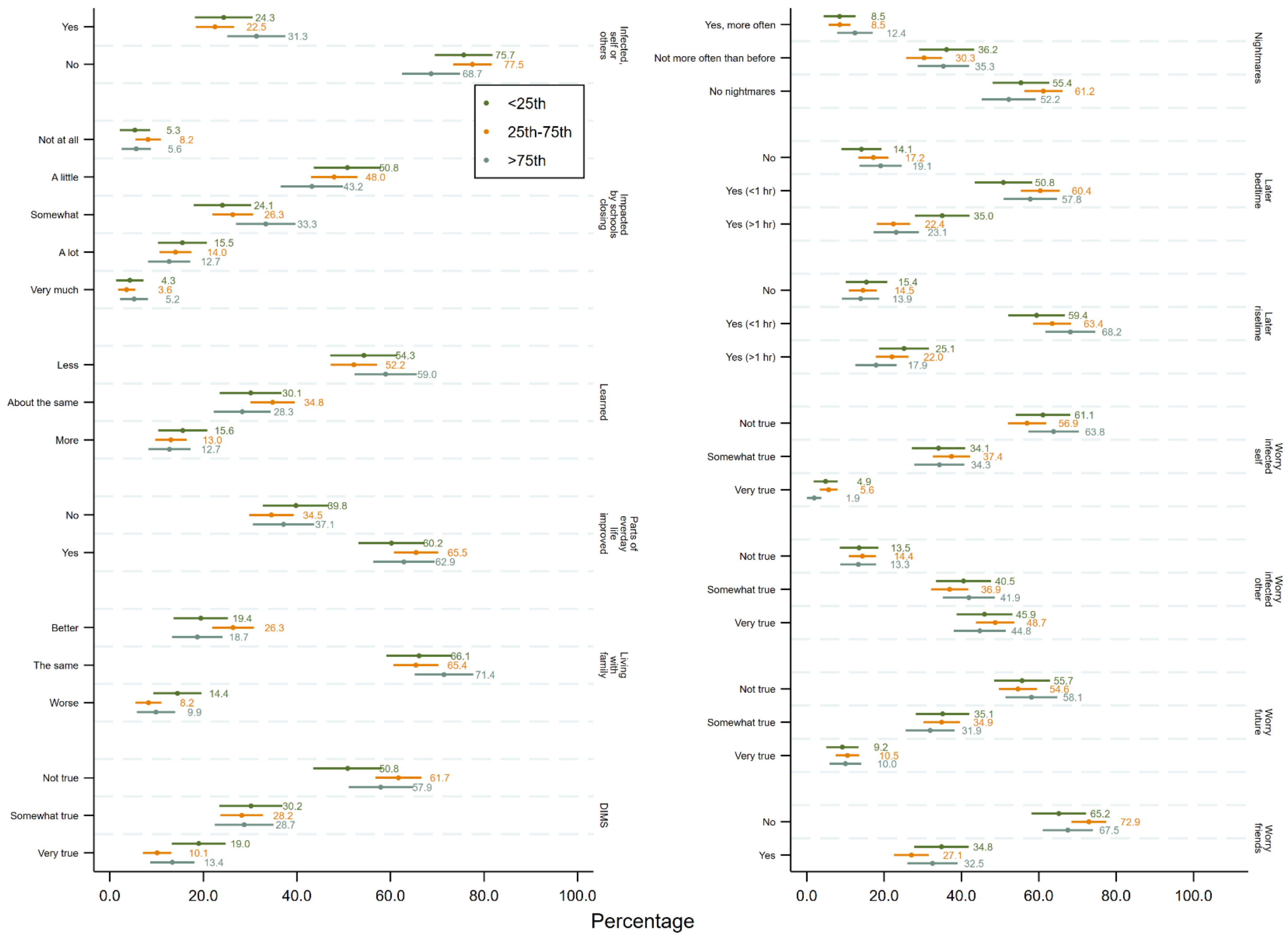


Supplementary Figure A3: Perceived consequences and worries across socioeconomic indicators. N=802. Bars denote 95% confidence intervals. Only Cohort 1: Parental-reported household income.

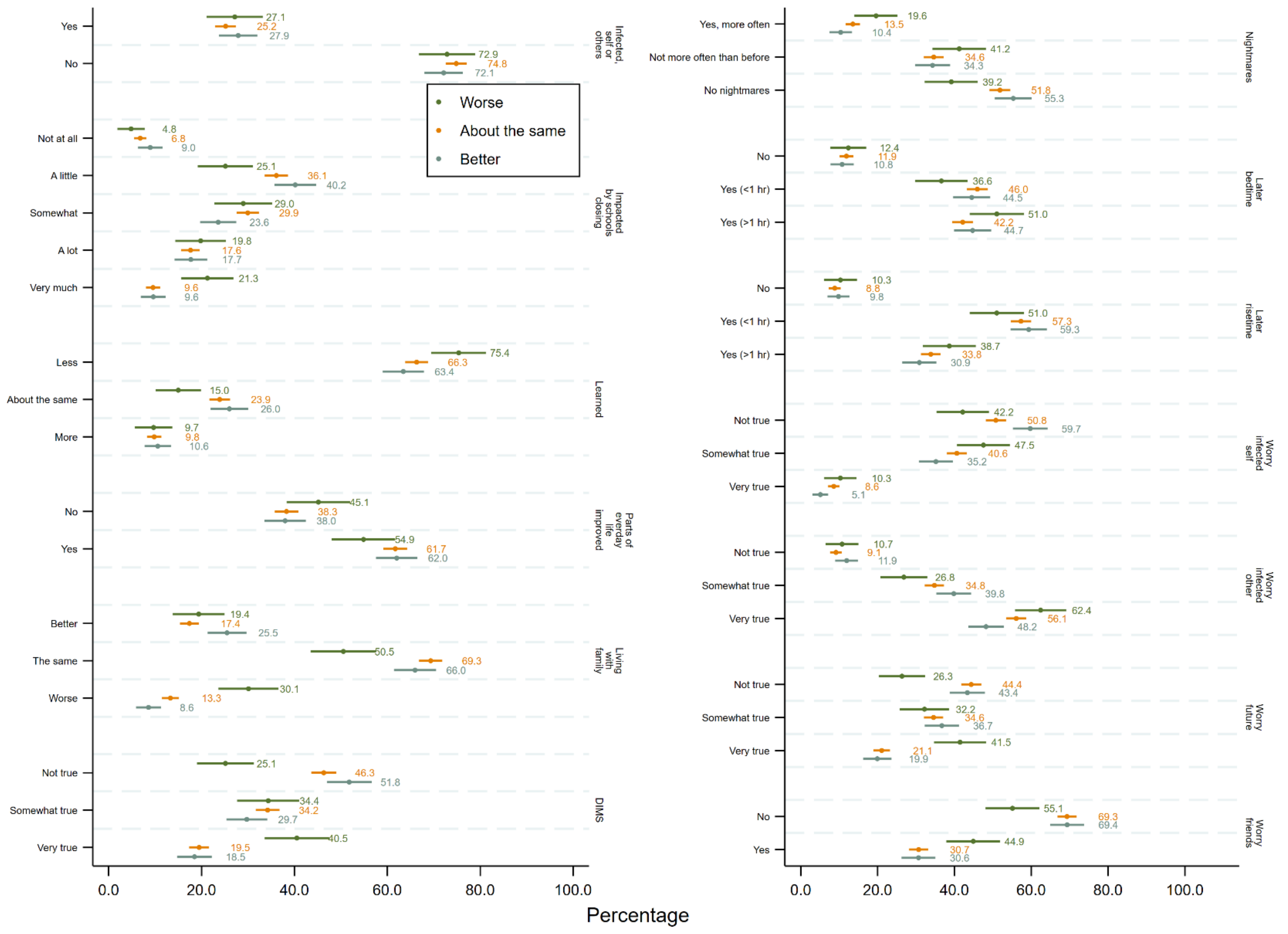


Supplementary Figure A4: Perceived consequences and worries across socioeconomic indicators. N=2091. Bars denote 95% confidence intervals. Only Cohort 2: Self-reported relative socioeconomic status.

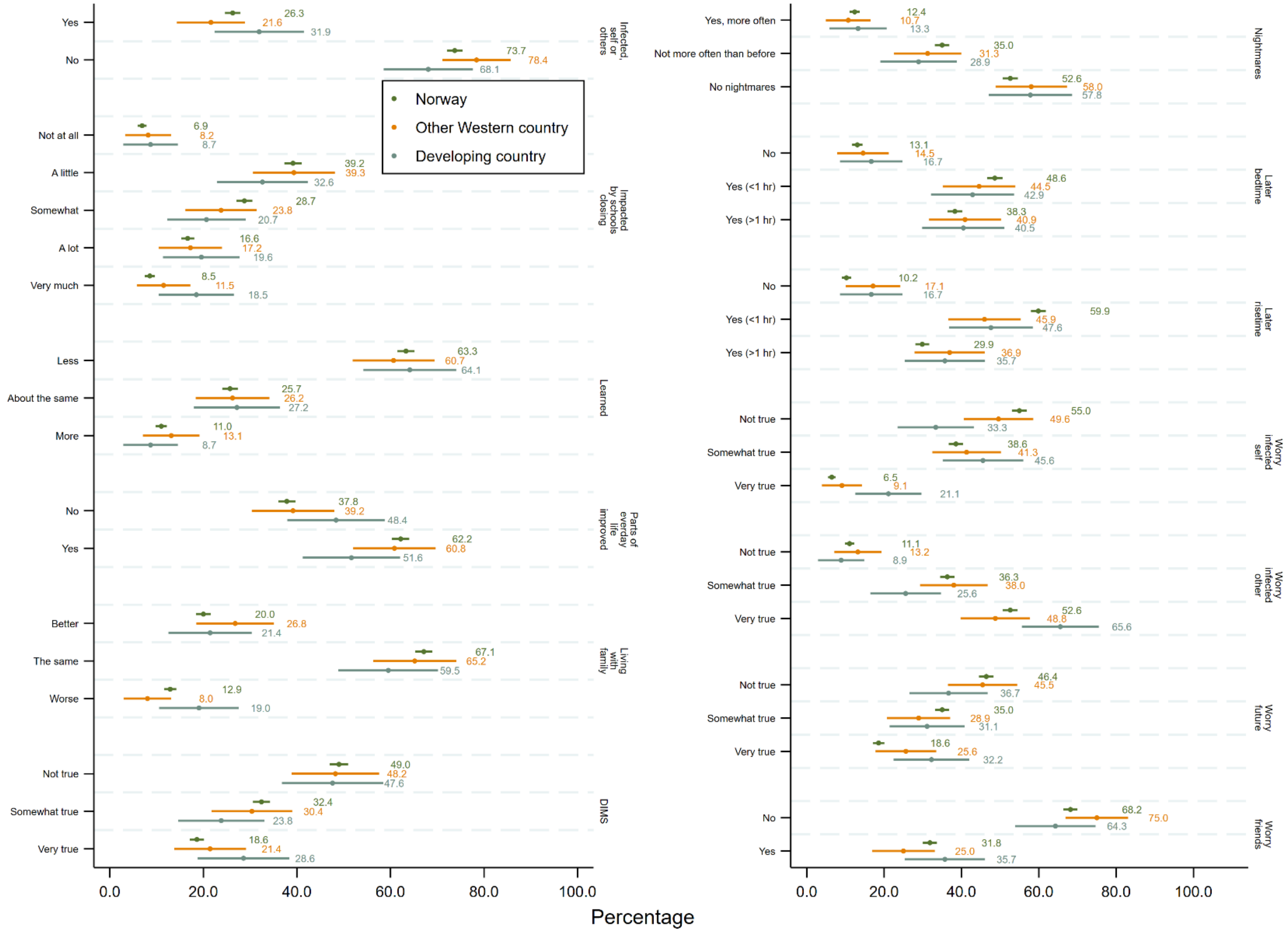


Supplementary Figure A5: Perceived consequences and worries across country of birth. N=2926. Bars denote 95% confidence intervals
